# Relationship between cirrhosis patients’ social support and post-traumatic stress symptoms: Chained multiple mediating effects of sense of control and avoidance coping strategies

**DOI:** 10.1097/MD.0000000000050156

**Published:** 2026-09-04

**Authors:** Jintao Cheng, Huiqiong Gui, Suyun Guo, Yihua Yang, Yun Ran

**Affiliations:** a Hepatology Department, Beijing University of Chinese Medicine Shenzhen Hospital (Longgang), Shenzhen, Guangdong Province, China.

**Keywords:** avoidance, chain mediation, liver cirrhosis, PTSS, sense of control, social support

## Abstract

Patients with cirrhosis frequently exhibit post-traumatic stress symptoms (PTSS), which may further aggravate serious psychological and psychiatric conditions. However, the underlying mediating mechanisms by which social support affects PTSS remain insufficiently elucidated. From November 2024 to July 2025, patients with cirrhosis were recruited via cluster sampling from a tertiary hospital in Shenzhen, Guangdong Province, China. A total of 380 participants completed the Perceived Social Support Scale, the civilian version of the Post-Traumatic Stress Checklist, the Sense of Control Scale, and the Self-Report Coping Scale. Data were analyzed using chi-square tests, Pearson’s correlation analyses, and structural equation modeling to examine the relationships among variables. The mean PTSS score was 29.09 ± 10.61, indicating a moderate level of symptom severity. Perceived social support (61.23 ± 15.53) and sense of control (49.22 ± 11.76) were relatively low, whereas avoidant coping subtypes – withdrawal (9.11 ± 4.64), internalization (8.95 ± 4.08), and externalization (10.86 ± 4.90) – were moderately prevalent. Structural equation modeling results indicated that perceived social support not only directly and negatively predicted PTSS, but also exerted significant indirect effects through sense of control, internalization, and externalization, collectively accounting for 25.21% of the total effect. Moreover, sense of control and internalization sequentially mediated the association between social support and PTSS, forming a significant chain mediation pathway. Social support mitigates PTSS both directly and indirectly via enhanced control and reduced avoidant coping (internalization/externalization). The identified chain mediation pathway underscores the importance of developing interventions that enhance sense of control and reduce maladaptive coping strategies in patients with cirrhosis.

## 1. Introduction

Liver cirrhosis is a progressive chronic liver disease characterized by diffuse hepatocyte necrosis, abnormal hepatocyte regeneration, angiogenesis, extensive fibrous tissue proliferation, and pseudolobular formation.^[1]^ It is mainly caused by alcoholic hepatitis, viral hepatitis, autoimmune hepatitis, and nonalcoholic fatty liver disease. Cirrhosis caused by various etiologies can progress to liver cancer.^[2]^ Global epidemiological data from 2017 report approximately 112 million cases of compensated cirrhosis and 10.6 million cases of decompensated cirrhosis, resulting in an estimated 1.32 million cirrhosis-related deaths.^[3]^ Mortality rates remain alarmingly high; for example, between 2002 and 2010, the fatality rate among Korean patients with cirrhosis was 30.66%, surpassing that of the 5 most prevalent cancers.^[4]^ China bears a disproportionate disease burden, with approximately 300 million cases of liver disease and accounting for 11% of global liver-related mortality.^[2,5]^

The disease exhibits an insidious progression: the compensated phase is typically asymptomatic, whereas the decompensated phase is characterized by portal hypertension and severe hepatic dysfunction, often complicated by life-threatening conditions such as ascites, gastrointestinal hemorrhage, and hepatic encephalopathy.^[6]^ Complications of liver cirrhosis usually have a sudden onset, dangerous condition, and high mortality rate. Patients may experience related psychological stress reactions such as anxiety, depression, and fear after undergoing rescue.^[5]^ In addition, the different causes and adverse outcomes of liver cirrhosis can have multiple impacts on the mental health of patients. After alcoholic hepatitis, liver cirrhosis is often accompanied by self-blame and depression.^[7]^ Patients with viral hepatitis are prone to feelings of shame and anxiety.^[8,9]^ The economic burden and life-threatening conditions brought by the deterioration of the condition (liver cancer) can trigger widespread anxiety and depression disorders,^[10]^ all of which affect the physical and mental health of patients. At the same time, stress reactions such as tension and anxiety can induce sympathetic nervous system excitation, increase heart rate and blood pressure, and also trigger cardiovascular and cerebrovascular diseases, posing a risk of further bleeding and affecting the recovery and prognosis of the disease.^[11]^ At the same time, stress reactions such as tension and anxiety can induce sympathetic nervous system excitation, increase heart rate and blood pressure, and also trigger cardiovascular and cerebrovascular diseases, posing a risk of further bleeding and affecting the recovery and prognosis of the disease.^[11]^

Post-traumatic stress symptoms (PTSS) – characterized by trauma-related avoidance, hypervigilance, and heightened arousal occurring within 1 to 30 days after trauma^[12]^ – constitute a significant health concern in this population. Early identification and intervention for PTSS are essential to safeguarding long-term psychological well-being. Social support – defined as the material and psychological resources derived from social relationships during periods of stress^[13]^ – can mitigate PTSS severity, whereas its absence is a robust predictor of adverse outcomes.^[14]^ According to Cohen’s Additive and Direct Model,^[15]^ social support buffers the psychological impact of trauma through mediating processes, potentially by enhancing coping strategies and an individual’s sense of control.

Avoidant coping strategies (e.g., denial, withdrawal) may offer short-term stress relief but are positively associated with PTSS development^[16]^ and long-term maladaptation.^[17]^ Social support facilitates the adoption of adaptive coping strategies, thereby reducing reliance on avoidant behaviors.^[18]^ A sense of control – the belief in one’s capacity to influence life events^[19]^ – functions as a cognitive resource that can be strengthened by social support.^[20]^ It plays a critical role in determining stress adaptability^[21]^: heightened control reduces helplessness and psychological distress,^[22]^ whereas diminished control increases vulnerability to PTSS.^[23]^ Cognitive theories further suggest that the persistence of PTSS arises from maladaptive appraisals of uncontrollability concerning trauma-related emotions and memories.^[24]^ Thus, a sense of control may exert a direct influence on PTSS and simultaneously reinforce avoidant coping strategies.

**Figure 1.**
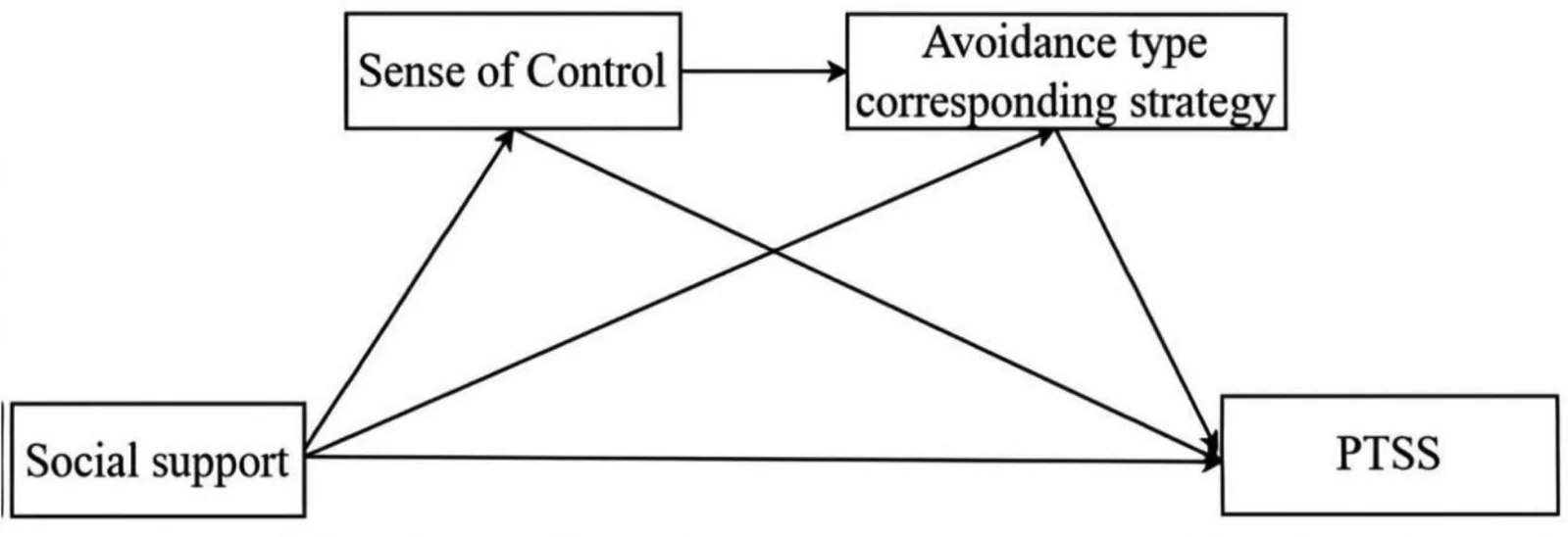
Theoretical framework of the study. This is a sequential chain mediation model. Based on this model, our research explores the sequential chain mediation effect of sense of control and avoidant coping style in the association between social support and PTSS of patients. PTSS= post-traumatic stress symptoms.

To elucidate these mechanisms, we propose a sequential chain mediation model (Fig. 1) to examine how a sense of control and avoidant coping strategies jointly mediate the relationship between social support and PTSS in patients with cirrhosis.

Building upon this theoretical framework, we examined the sequential chain mediation effects of sense of control and avoidant coping in the association between social support and PTSS among patients with liver cirrhosis. Based on this framework, the following hypotheses were proposed:

H1: Social support is negatively associated with PTSS severity in patients with liver cirrhosis.
H2: Sense of control mediates the association between social support and PTSS.
H3: Avoidant coping strategies mediate the association between social support and PTSS.
H4: Sense of control and avoidant coping strategies sequentially mediate the association between social support and PTSS.

## 2. Materials and methods

### 2.1. Participants

Using a convenience sampling approach, patients with cirrhosis were recruited from both outpatient and inpatient units of a tertiary public hospital in Shenzhen, Guangdong Province, China, from November 2024 to July 2025. Inclusion criteria were as follows: confirmed diagnosis of compensated or decompensated cirrhosis; age ≥ 18 years; ability to complete questionnaires independently or with assistance from research staff; and provision of written informed consent. Exclusion criteria included: severe chronic comorbidities (e.g., cardiac, pulmonary, or renal failure); and cognitive impairment or psychiatric disorders that could interfere with questionnaire completion.

### 2.2. Data collection

**Figure 2.**
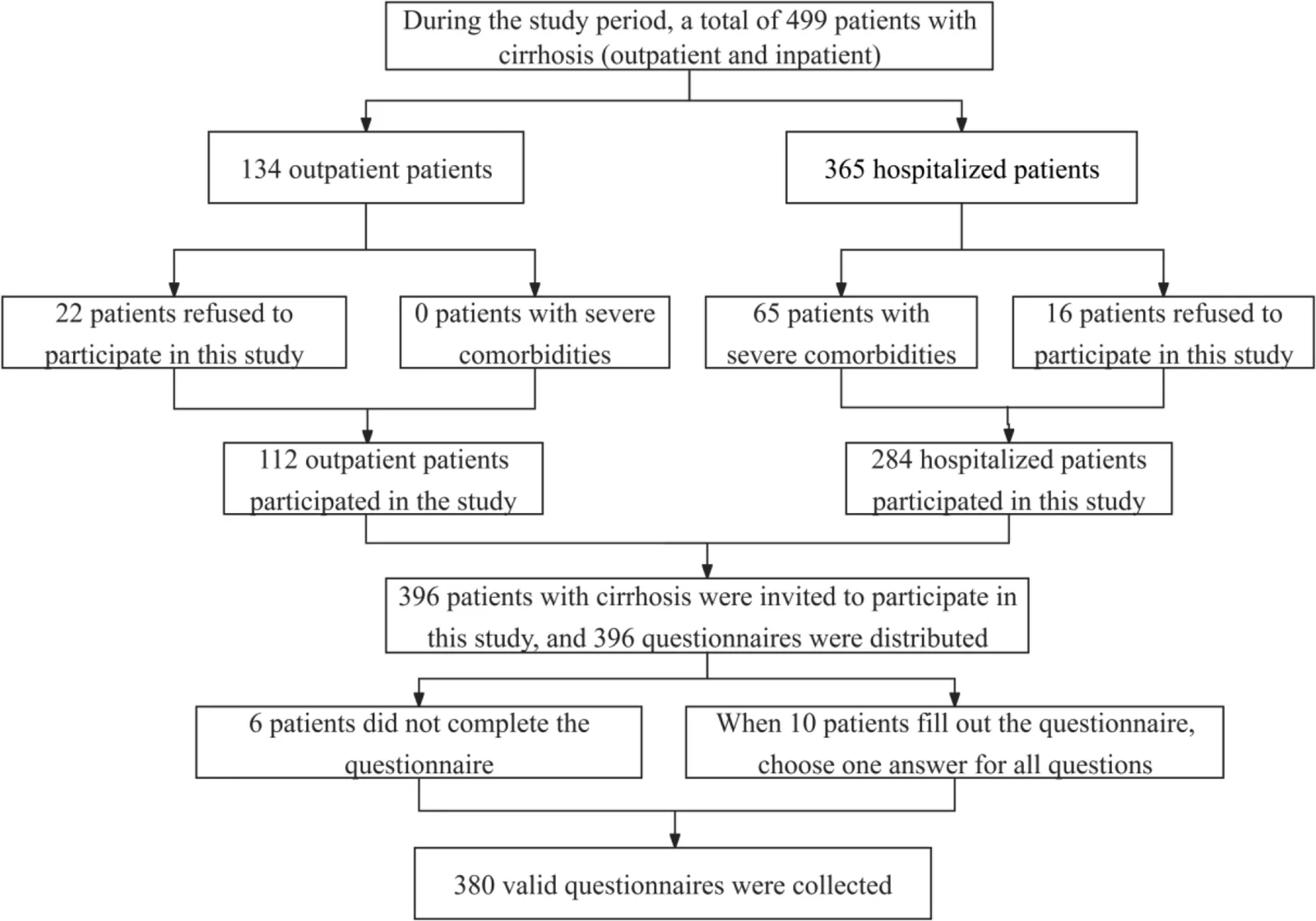
Patient inclusion process diagram. During the study period, 499 patients with liver cirrhosis were invited to participate in this study, including 134 outpatients and 365 inpatients. We excluded patients who were unwilling to participate in this study, had serious complications and could not complete the questionnaire survey. This study really investigated 380 patients with liver cirrhosis.

Data were collected through both online questionnaires (administered via Wenjuanxing: https://www.wjx.cn) and paper-based formats. Trained research staff provided standardized explanations of the study’s objectives, procedures, and significance to all participants. After providing written informed consent, participants completed the questionnaires anonymously and independently. Research staff checked the completeness of the questionnaires on site, with an average completion time of approximately 10 minutes. Questionnaires containing excessive missing data or exhibiting patterned responses were excluded from analysis. Of the 396 patients invited to participate, 16 were excluded (6 due to incomplete responses and 10 due to invalid patterned answers), resulting in 380 valid questionnaires, corresponding to a response rate of 95.96%. Among these, there were 89 paper questionnaires (23.4%) and 291 electronic questionnaires (76.6%). The specific process of patient inclusion is shown in Figure 2.

### 2.3. Research tools

#### 2.3.1. General Information Questionnaire

A comprehensive literature review was conducted to identify demographic and clinical variables relevant to patients with liver cirrhosis. Based on the literature review, the demographic and clinical variables collected in this study comprised age, occupation, sex, education level, marital status, type of medical insurance, income, and stage of liver cirrhosis.

#### 2.3.2. Perceived Social Support Scale (PSSS)

The Perceived Social Support Scale (PSSS) was originally developed by Zimet et al and subsequently adapted into a Chinese version by Jin .^[25]^ The scale comprises 4 dimensions – family support, friend support, and significant-other support – which assess the extent of perceived support from these respective sources. The total score reflects the individual’s overall perceived level of social support. The scale contains 12 items, each rated on a 7-point Likert scale. Higher total scores indicate greater levels of perceived social support. In the present study, the Cronbach’s alpha coefficient for the total scale was 0.946, with coefficients of 0.855, 0.859, and 0.857 for the dimensions of family support, friend support, and significant-other support, respectively.

#### 2.3.3. PTSS Civilian Version (PCL-C)

The Post-traumatic Stress Disorder Checklist – Civilian Version (PCL-C) was developed by the US National Center for Post-traumatic Stress Disorder and subsequently translated into Chinese by Chao and co-workers.^[26]^ It is designed to assess trauma-related symptoms experienced by individuals in their daily lives. The scale comprises four dimensions: reexperiencing, numbness, avoidance, and hyperarousal. It contains 17 items, each rated on a 5-point Likert scale, with higher total scores indicating greater severity of PTSS-related symptoms. A total score of 17 to 37 indicates no significant PTSS; 38 to 49 suggests mild-to-moderate PTSS; and 50 to 85 reflects severe PTSS.^[27]^ In the present study, the Cronbach’s alpha coefficient for the total scale was 0.947, with coefficients of 0.836, 0.884, and 0.831 for the dimensions of reexperiencing, avoidance/numbness, and hyperarousal, respectively.

#### 2.3.4. Spheres of Control Scale (SCS)

The Spheres of Control Scale, developed by Paulhus in 1983, is designed to measure an individual’s sense of control over personal behavior, interpersonal relationships, and sociopolitical contexts.^[28]^ The scale contains 30 items divided into three subscales: personal efficacy, interpersonal control, and sociopolitical control. In the present study, only the Personal Efficacy subscale was used, comprising 10 items rated on a 7-point Likert scale. Higher scores indicate a stronger perceived sense of control over personal planning, achievement, and related aspects. In this study, the Cronbach’s alpha coefficient for the Personal Efficacy subscale was 0.920.

#### 2.3.5. Self-Report Coping Scale (SRCS)

The scale, revised by Kochenderfer-Ladd and Skinner,^[29]^ consists of 22 items categorized into 5 dimensions: help-seeking, problem-solving, internalization, externalization, and withdrawal. It utilizes a 5-point Likert scale, with higher scores indicating greater use of the corresponding coping strategy. The present study exclusively examines avoidance coping strategies, specifically internalization, externalization, and withdrawal. The overall Cronbach’s α for these three dimensions was 0.843, with individual coefficients of 0.906 for internalization, 0.884 for externalization, and 0.891 for withdrawal.

### 2.4. Ethical principles

This study was approved by the institutional ethics committee (approval number: KY-2024-239-01), and all procedures strictly adhered to the principles of informed consent, non-maleficence, and confidentiality throughout the research process. The questionnaire incorporated an informed consent form and an explicit consent option for participants, who were required to select the “informed consent” option prior to answering the questionnaire items. Moreover, participants were assured that withdrawing from the study would have no adverse consequences. To ensure the accuracy and authenticity of responses, the questionnaire was administered anonymously. All collected data were used exclusively for this study and were subject to strict confidentiality protocols.

### 2.5. Statistical analysis

Descriptive statistics and correlation analyses were conducted using SPSS version 29.0 (International Business Machines Corporation), whereas structural equation modeling (SEM) and mediation effect testing were performed using AMOS version 24.0 (International Business Machines Corporation).

#### 2.5.1. Testing of mediating effects

The product-of-coefficients test was employed to assess mediating effects. Compared with the causal steps approach, this method does not infer the presence or absence of mediation through a sequence of hypothesis tests. Instead, it directly evaluates whether the mediation effect (ab) is significant and nonzero, and provides point estimates as well as confidence intervals for mediation effects, thereby offering higher statistical power.^[30]^ Based on SEM, the present study applied the bias-corrected nonparametric percentile bootstrap within the product-of-coefficients framework to examine the mediating effect.

#### 2.5.2. Model fit evaluation criteria

The evaluation of structural equation model fit involves three categories of indices: absolute fit indices, relative fit indices, and parsimonious fit indices. Specifically, χ^2^/d*f*, goodness-of-fit index (GFI), adjusted goodness-of-fit index (AGFI), and root mean square error of approximation (RMSEA) are classified as absolute fit indices; comparative fit index (CFI), TLI, normed fit index (NFI), and incremental fit index (IFI) as relative fit indices; and PGFI as a parsimonious fit index. In general, a χ^2^/d*f* ratio between 1 and 3 suggests an acceptable model fit; however, this index is sensitive to sample size fluctuations. When the sample size is large, model fit should be evaluated in conjunction with other indices. An RMSEA value less than or equal to 0.10 indicates acceptable fit, whereas values of GFI, AGFI, CFI, TLI, NFI, and IFI > 0.90 are generally considered indicative of good fit.^[24]^

## 3. Results

### 3.1. Common method bias

To evaluate potential artificial covariance between predictor and criterion variables arising from the same data source, measurement environment, item context, and item characteristics, Harman’s single-factor test was applied to assess common method bias. An exploratory factor analysis without rotation was conducted on all items from the PSSS, PTSS Civilian Version, Control Circle Scale, and Self-Reported Coping Scale using SPSS version 29.0. The analysis extracted 6 factors with eigenvalues >1, with the first factor accounting for 21.78% of the variance – well below the critical threshold of 50%.^[31]^ These results indicate that common method bias was not a significant concern in this study.

### 3.2. General characteristics of participants

Among the 380 patients with cirrhosis included in the analysis, 256 (67.4%) were male and 124 (32.6%) were female. In terms of marital status, 26.6% were unmarried, 71.1% were married, and 2.4% were divorced. The majority of patients (78.7%) were aged between 30 and 60 years. Approximately three-fifths (59.8%) reported a monthly income exceeding 5000 yuan, whereas 16.8% had an income below 2000 yuan. Educational attainment was generally high, with 63.5% holding a bachelor’s degree or above. Unemployment was reported by 9.7% of participants, 75.3% are hospitalized patients, 56.1% of patients with post viral hepatitis cirrhosis and 52.9% of patients with decompensated cirrhosis. Please refer to Table 1 for detailed data. Subgroup analyses based on disease-stage (compensated vs decompensated) were not performed due to limited statistical power, as the low overall PTSS prevalence (7.89%) precluded meaningful stratified comparisons.

**Table 1:** Detailed demographic information is presented.

Variable		Number	Rate (%)
Gender	Male	256	67.4
	Female	124	32.6
Age	<18 yr old	5	1.3
	18–30 yr old	54	14.2
	30–40 yr old	115	30.3
	40–50 yr old	132	34.7
Age	50–60 yr old	52	137
	≥60 yr old	22	5.8
Occupation	Farmer	26	6.8
	Retiree	20	5.3
	Institution	90	23.7
	Unemployment	37	9.7
	Staff	60	15.8
	Liberal professions	82	21.6
	Worker	65	17.1
Marriage	Married	270	71.1
	Unmarried	101	26.6
	Divorce	9	2.4
Income	<2000¥	64	16.8
	2000–5000¥	89	23.4
	5000–7000¥	175	46.1
	≥7000¥	52	13.7
Educational	Junior college or below	139	36.6
	Undergraduate	213	56.1
	Master degree or above	28	7.4
Medical insurance	Yes	312	82.1
	No	68	17.9
Patient-derived	Outpatient service	96	25.3
	Inpatient department	284	74.7
Cirrhosis staging	Compensatory stage	179	47.1
	Decompensation stage	201	52.9
Cause of disease	Viral hepatitis	213	56.1
	Alcoholic Hepatitis	86	22.6
	NAFLD	42	11.1
	Others	39	10.3

### 3.3. Social support, sense of control, avoidance coping strategies, and PTSS levels in patients with cirrhosis

In this study, the mean social support score among patients with cirrhosis was 61.23 ± 15.53, which is below the median reference score of 65 for the scale. The mean PTSS score was 29.09 ± 10.61, lower than the diagnostic cutoff score of 38, The scores for the three dimensions of reeexperience, avoidance/numbness, and excessive arousal are 8.69 ± 3.30 points, 10.33 ± 3.99 points, and 10.17 ± 3.85 points, respectively, which are relatively high compared to their median values. The mean sense of control score was 49.22 ± 11.76, which is below the median of 52 for the scale. The mean avoidance score was 9.11 ± 4.64, slightly higher than the median score of 8. The mean internalization score was 8.95 ± 4.08, representing a moderate level compared with the median of 8, while the mean externalization score was 10.86 ± 4.90, also at a moderate level compared with the median of 9. Among the 380 patients surveyed, 350 (92.11%) exhibited no significant PTSS, 19 (5.00%) had mild PTSS, and 11 (2.89%) met the criteria for significant PTSS, resulting in a positive screening rate of 7.89%.

### 3.4. Correlation between PTSS, social support, sense of control, and avoidance coping strategies

Previous studies suggest that PTSS in patients with cirrhosis may be influenced by demographic variables such as gender and age. Therefore, in the present analysis, gender and age were included as control variables, and partial correlation analyses were conducted for each variable (Table 2).

**Table 2:** Results of correlation analysis between research variables.

	Social support	PTSS	Sense of control	Avoidance	Internalization	Externalization
Social support	1					
PTSS	−0.213**	1				
Sense of control	0.215**	−0.228**	1			
Avoidance	0.008	0.038	−0.136*	1		
Internalization	−0.220**	0.233**	−0.210**	0.207**	1	
Externalization	0.101*	0.122*	−0.122*	0.118*	0.191**	1

PTSS = post-traumatic stress symptoms.

* Indicates *P* < .05 level (dual tailed).

** Indicating *P* < .01 (double tailed).

The results indicated that PTSS was significantly negatively correlated with social support and sense of control, significantly positively correlated with internalization and externalization, and not significantly correlated with avoidance. Social support was significantly positively correlated with sense of control and externalization, significantly negatively correlated with internalization, and not significantly correlated with avoidance. Sense of control was significantly negatively correlated with internalization, externalization, and avoidance.

These findings suggest the potential mediating roles of sense of control, as well as internalizing and externalizing coping strategies, in the relationship between social support and PTSS. Additionally, since controlling for gender and age did not substantially alter the correlation coefficients, these demographic variables were not included in the subsequent mediation model analysis.

### 3.5. Prediction of PTSS by social support: the mediating role of sense of control and avoidant coping strategies

#### 3.5.1. Structural equation modeling

A structural equation model (SEM) was constructed with social support as the independent variable, PTSS as the dependent variable, and sense of control together with internalizing and externalizing coping strategies as mediating variables. To compare different model specifications, 5 mediation models were successively established: the complete mediation model (M1), the partial mediation model (M2), the single-chain mediation model (M3), the double-chain mediation model (M4), and the multi-stage chain model (M5).

M1 (Complete Mediation Model): social support influences PTSS entirely through the three mediators – sense of control, internalization, and externalization. M2 (Partial Mediation Model): based on M1, a direct path from social support to PTSS is added. M3 (Single-Chain Mediation Model): based on M2, a direct path from sense of control to internalization is added. M4 (Double-Chain Mediation Model): based on M3, a direct path from sense of control to externalization is added. M5 (Multi-Stage Chain Model): based on M3, direct paths between internalization and externalization are added.

The rationale for comparing these 5 models was to systematically evaluate whether the data supported increasingly complex mediation pathways. Specifically, M1 tested whether the effect of social support on PTSS was fully mediated; M2 examined whether a direct effect remained after accounting for mediators; M3 and M4 assessed whether sense of control sequentially influenced specific avoidant coping strategies; and M5 tested whether internalization and externalization operated in a sequential manner, thereby representing the most theoretically comprehensive chain mediation structure.

Model fit was evaluated using the maximum likelihood method, and the results are presented in Table 3. Given that the χ^2^ statistic tends to increase rapidly with sample sizes between 200 and 500, the χ^2^/df ratio in this study was used for reference only. As shown in Table 3, the RMSEA of the multi-stage chain mediation model (M5) was 0.10, and all other fit indices (GFI, AGFI, CFI, TLI, NFI, and IFI) exceeded the 0.90 threshold.^[24]^

**Table 3:** Model adaptation index of the effects of perceived and avoidant coping strategies on the relationship between social support and PTSS.

Model	χ^2^	d*f*	χ^2^/d*f*	RMSEA	GFI	AGFI	CFI	IFI	NFI
M1	44.704	4	11.176	0.164	0.953	0.822	0.639	0.657	0.636
M2	35.092	3	11.676	0.168	0.962	0.809	0.716	0.732	0.715
M3	23.836	2	11.918	0.170	0.976	0.821	0.806	0.819	0.806
M4	15.492	1	15.492	0.196	0.984	0.763	0.871	0.881	0.874
M5	4.997	1	4.997	0.100	0.995	0.922	0.965	0.967	0.959

AGFI = adjusted goodness-of-fit index, CFI = comparative fit index, GFI = goodness-of-fit index, IFI = incremental fit index, NFI = normed fit index, PTSS = post-traumatic stress symptoms, RMSEA = root mean square error of approximation.

**Figure 3.**
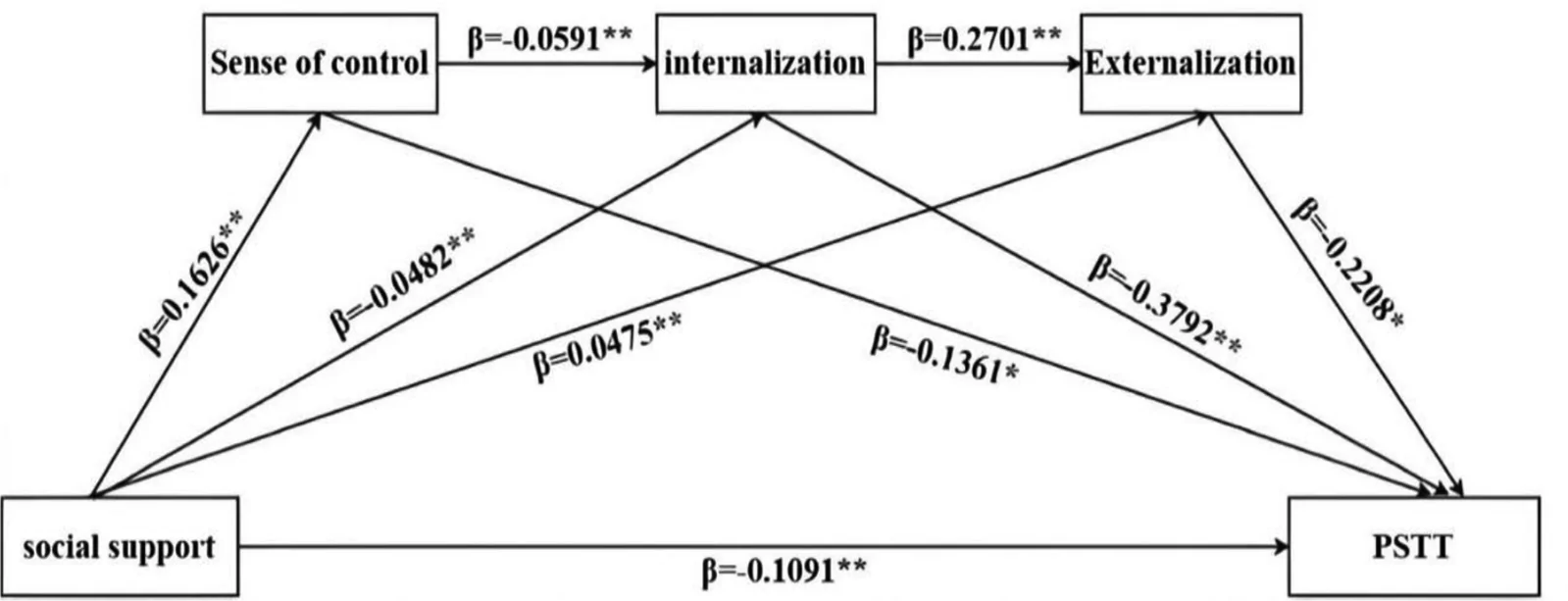
Chain mediation model of the relationship between social support and PSTT using control and avoidance coping strategies. This model indicates that social support exerts both a direct effect on PTSS and an indirect effect through 6 mediation pathways: Social support → sense of control → PTSS, Social support → internalization → PTSS, Social support → externalization → PTSS, Social support → sense of control → internalization → PTSS, Social support → internalization → externalization → PTSS, Social support → sense of control → internalization → externalization → PTSS. PTSS = post-traumatic stress symptoms.

Based on these results, the multi-stage chain mediation model (M5) was selected (Fig. 3). This model indicates that social support exerts both a direct effect on PTSS and an indirect effect through 6 mediation pathways:

1. Social support → sense of control → PTSS.
2. Social support → internalization → PTSS.
3. Social support → externalization → PTSS.
4. Social support → sense of control → internalization → PTSS.
5. Social support → internalization → externalization → PTSS.
6. Social support → sense of control → internalization → externalization → PTSS.

#### 3.5.2. Significance test of mediation effect

The Bootstrap test results indicate that the 95% CI of all mediation pathways does not include 0, which is statistically significant, indicating a significant mediation effect, as shown in Table 4. In summary, social support directly affects PTSS and indirectly affects PTSS through 6 mediating pathways consisting of control and internalization/externalization coping strategies. The mediating effect accounts for 25.21% of the total effect size (−0.14587).

**Table 4:** The results of the significance test for the mediating effect.

Effect type	Route	Effect	95% CI		*P*
			Lower	Upper	
direct effect	Social support → PTSS	−0.10911	−0.19325	−0.03119	.00680
indirect effect	Social support→sense of control→PTSS	−0.02213	−0.05347	−0.00476	.00447
	Social support→internalization→PTSS	−0.01829	−0.05471	−0.00154	.02565
	Social support→externalization→PTSS	0.01086	0.00128	0.02831	.02355
	Social support →sense of control→ internalization→PTSS	−0.00364	−0.01336	−0.00035	.02026
	Social support → internalization → externalization → PTSS	−0.00298	−0.01067	−0.00027	.02185
	Social support → sense of control → internalization → externalization → PTSS	−0.00059	−0.00313	−0.00004	.02081
Total effects		−0.14587	−0.25456	−0.04851	.00300

CI = confidence interval, PTSS = post-traumatic stress symptoms.

## 4. Discussion

Given the cross-sectional and single-center nature of this study, all reported associations should be interpreted as correlational rather than causal. The directional pathways proposed in the mediation model are theoretically driven rather than empirically verified as causal sequences. Accordingly, the term “effects” is used throughout this discussion in a statistical, not causal, sense.

### 4.1. Levels and current status of PTSS in patients with cirrhosis

The findings of this study indicate that the majority of patients with cirrhosis did not exhibit significant PTSS, with only 7.89% testing positive on screening. This prevalence is markedly lower than that reported in certain populations experiencing major trauma or severe illness.^[32,33]^ Overall, the mean PTSS score was 29.09 ± 10.61, which is below the commonly accepted clinical diagnostic cutoff of 38, suggesting a relatively mild overall psychological trauma response among the study cohort. However, in terms of symptom dimensions, scores for reexperiencing, avoidance/numbing, and hyperarousal (8.69 ± 3.30, 10.33 ± 3.99, and 10.17 ± 3.85, respectively) were all above their median levels, indicating that, despite low overall scores, some patients exhibited pronounced psychological distress in specific symptom domains. This pattern of “overall mild but locally elevated” suggests that, in clinical management, attention should be given to patients who do not meet PTSS diagnostic criteria but demonstrate notable reactions in core symptom clusters, enabling early identification of potential mental health risks and the implementation of targeted interventions.

International studies have reported that the prevalence of PTSS among patients with liver disease, particularly those awaiting liver transplantation, can reach 15% to 25%, markedly exceeding the 7.89% observed in the present study.^[26,27]^ This discrepancy may be attributable to more severe disease conditions, more invasive treatment modalities, and cultural differences in illness perception in those populations. National surveys have similarly reported detection rates of PTSS in patients with chronic diseases ranging from 5% to 12%,^[34,35]^ which is consistent with the findings of this study. However, scores in core symptom dimensions such as reexperiencing and hyperarousal are generally lower in these populations. In contrast, the present study found both dimensions to be above median levels, suggesting that patients with cirrhosis may be particularly sensitive to disease-related traumatic memories during certain life events or disease stages. Furthermore, compared with populations such as patients with malignant tumors or those discharged from intensive care units, the overall PTSS level in this cohort was significantly lower.^[36,37]^ This may be related to the relatively slow progression of cirrhosis, less frequent exposure to traumatic events, and the gradual development of adaptive psychological mechanisms. These differences not only underscore the complex interplay between disease type, disease course, and psychological response patterns but also highlight the need for tailored risk screening and intervention strategies based on the symptom spectrum and cultural context of specific patient groups. The low prevalence (7.89%) also implies that the present sample may have been underpowered for detecting clinically meaningful subgroup differences (e.g., compensated vs decompensated), and future studies with larger samples are warranted to examine such stratification.

### 4.2. Relationship between PTSS, social support, sense of control, and avoidance coping strategies in patients with cirrhosis

This study found that social support was negatively correlated with PTSS in patients with cirrhosis, consistent with Cohen and Syme’s^[38]^ social support model, which posits that social support mitigates trauma-induced stress by influencing individuals’ cognitive and emotional states. According to the stress system theory model,^[39]^ psychological stress outcomes are shaped by both internal and external factors – including social support, cognitive appraisal, and coping styles – that interact dynamically. Social support, as a network providing both material and emotional assistance, plays a critical buffering role in the stress response process.^[40-42]^ Greater social support equips patients with more resources to withstand traumatic events, thereby preventing or alleviating PTSS.

A significant negative association was also observed between PTSS and sense of control, indicating that patients with a stronger sense of control experienced fewer PTSS symptoms. This finding aligns with Lazarus et al’s stress theory,^[43]^ which suggests that sense of control over one’s life reduces stress responses, whereas lack of control can exacerbate PTSS. Enhancing patients’ sense of control – particularly in disease management and daily life – may therefore be an effective clinical strategy to mitigate PTSS by increasing confidence, self-efficacy, and adaptive capacity in the face of trauma.

Conversely, PTSS was positively associated with internalization, externalization, and withdrawal coping strategies. While avoidance strategies may temporarily reduce emotional burden, they hinder the confrontation of trauma and the adoption of effective solutions, ultimately exacerbating psychological distress.^[44-46]^ Roth and Cohen ^[47]^ noted that avoidance may provide short-term relief but carries drawbacks such as emotional numbing, lack of symptom awareness, and potential maladaptive behaviors. Thus, persistent reliance on avoidance strategies increases PTSS risk.

### 4.3. Mediating role of sense of control and avoidance coping strategies

The results of SEM in this study indicate that sense of control, internalization, and externalization serve as mediators between social support and PTSS, suggesting that social support exerts both direct and indirect effects on PTSS through these variables.

Sense of control demonstrated a significant mediating effect between social support and PTSS. Social support indirectly mitigates PTSS primarily by enhancing patients’ sense of control, which, as a critical psychological resource, reflects an individual’s perceived capacity to regulate their life and manage disease. This finding aligns with the learned helplessness theory^[21]^ and stress theory, both of which posit that increased control enhances an individual’s ability to adapt to stressful events, thereby reducing helplessness and psychological distress. By strengthening patients’ sense of control, social support promotes the adoption of proactive coping strategies and reduces the likelihood of PTSS. Previous research has demonstrated that an enhanced sense of control can mitigate perceived helplessness and passivity following trauma, thereby effectively reducing PTSS.^[37]^ The present study further corroborates this relationship, indicating that stronger social support is associated with a higher sense of control, which in turn corresponds to lower PTSS levels. This is consistent with the social regulation model proposed by Valenzuela et al,^[48]^ who argued that social support can enhance self-efficacy through cognitive regulation pathways, thereby buffering the onset of trauma-related symptoms. Moreover, sense of control is significantly correlated with avoidant coping strategies (internalization and externalization), underscoring its central role in the chain mediation model. When patients with cirrhosis perceive greater control over their lives, they exhibit higher confidence and self-efficacy, enabling more adaptive responses to traumatic events.

Avoidant coping strategies – specifically internalization and externalization – also exert significant mediating effects between social support and PTSS. This observation is consistent with Weiss et al,^[16]^ who suggested that avoidant coping may temporarily relieve stress but ultimately exacerbate PTSS symptoms over time. In this study, social support indirectly reduced the risk of PTSS by lowering patients’ reliance on avoidant coping. Specifically, social support negatively predicted internalization, whereas internalization positively predicted PTSS severity. These findings indicate that maladaptive coping – especially internalization – intensifies emotional responses to trauma, thereby worsening PTSS, consistent with Patronick et al.^[45]^ Although externalization showed a weak positive correlation with social support, it still positively predicted PTSS, suggesting that externalization fails to alleviate trauma burden, consistent with Hyun.^[49]^

Furthermore, this study identified a chain mediation pathway – “sense of control → internalization → externalization” – through which social support indirectly influences PTSS. This finding suggests that psychological resources (sense of control) exert a regulatory effect on behavioral responses (avoidance strategies), and that different avoidance strategies may interact synergistically. This mechanism is supported by Shapira et al,^[44]^ who reported in a sample of Israeli youth that sense of control significantly impacts emotion regulation strategies, which in turn predict PTSS severity. The present study extends these findings to patients with chronic physical illnesses, highlighting the importance of enhancing both psychological resources and behavioral coping mechanisms in interventions. Overall, social support affected PTSS through 6 mediating pathways, with indirect effects accounting for 25.21% of the total effect, underscoring the multidimensional nature of the underlying psychological mechanisms. These results support the “additional direct” dual-pathway model of social support, in which social support alleviates symptoms both directly and indirectly via cognitive and coping resources.

In summary, this study examined the association between social support and PTSS, and verified the mediating roles of sense of control and avoidance coping strategies. Cirrhosis may induce substantial psychological trauma, and social support constitutes a critical factor enabling patients to cope effectively with the disease and to mitigate the development of PTSS. To maximize the protective effects of social support, it is essential to assist patients in understanding, evaluating, and optimally utilizing both internal and external resources; to strengthen their sense of control over disease-related events and self-management; and to guide the appropriate use of coping strategies, particularly by minimizing the potential adverse effects associated with avoidance coping.

### 4.4. Limitations and future directions

As a cross-sectional study conducted at a single tertiary center in Shenzhen (n = 380), this research is subject to several methodological limitations that warrant consideration. First, the single-center design and moderate sample size may constrain the generalizability of findings to broader cirrhotic populations, particularly those in primary care settings or rural areas. Second, while clinical evidence suggests that decompensated patients face elevated PTSS risks due to recurrent hospitalization episodes and acute complications,^[50]^ the lack of disease-stage subgroup analysis – due to limited statistical power given the low PTSS prevalence (7.89%) – potentially obscures risk stratification. Future studies with larger, more diverse samples should include a priori planned subgroup analyses to examine whether the mediation pathways differ between compensated and decompensated patients. Third, the PCL-C scale, though validated for general trauma assessment, may exhibit limited sensitivity to cirrhosis-specific traumatic experiences (e.g., variceal bleeding episodes or transplant waitlist anxiety).^[51]^ The low PTSS prevalence may also reflect the use of a generic trauma measure that does not fully capture disease-specific distress. Fourth, the analysis was restricted to avoidance coping mediators, excluding other potentially relevant psychological constructs (e.g., resilience or cognitive reappraisal). Fifth, reliance solely on self-report measures, while partially addressed by Harman’s single-factor test, cannot fully eliminate concerns regarding common method variance; future studies should consider incorporating clinician-administered interviews or informant reports to triangulate findings. To address these limitations, future studies should: employ multicenter longitudinal designs to establish temporal relationships between social support and PTSS trajectories; incorporate disease-specific trauma measures (e.g., PCL-Medical version) with staged subgroup analyses; examine a broader range of mediators, including biological markers (e.g., cortisol levels) and positive coping mechanisms; and include objective clinical indicators (e.g., disease severity scores, laboratory values) to complement self-report data. Such advancements would strengthen the theoretical framework and clinical applicability of these findings, particularly for high-risk subgroups like decompensated cirrhosis or posttransplant patients.

## Acknowledgments

We would like to express our special thanks to all the liver cirrhosis patients who participated in this study, as well as all the medical staff from the Hepatology Department of Beijing University of Traditional Chinese Medicine Shenzhen Hospital (Longgang) for their support and assistance.

## Author contributions

**Data curation:** Jintao Cheng, Huiqiong Gui, Suyun Guo, Yihua Yang.

**Formal analysis:** Huiqiong Gui, Suyun Guo, Yihua Yang, Yun Ran.

**Funding acquisition:** Jintao Cheng, Huiqiong Gui.

**Investigation:** Jintao Cheng, Suyun Guo.

**Methodology:** Jintao Cheng.

**Visualization:** Yihua Yang.

**Writing – original draft:** Jintao Cheng, Huiqiong Gui, Yihua Yang, Yun Ran.

**Writing – review & editing:** Huiqiong Gui, Suyun Guo, Yihua Yang, Yun Ran.
